# Patient stratification for dose scaling in cervical cancer: a model-based analysis using image-guided adaptive brachytherapy

**DOI:** 10.1093/jrr/rrag047

**Published:** 2026-07-07

**Authors:** Tenyoh Suzuki, Ryo Takahashi, Hidenobu Tachibana, Shioto Oda, Takeshi Fujisawa, Masaki Nakamura, Hidehiro Hojo, Kento Tomizawa, Weishan Chang, Tetsuo Akimoto, Sadamoto Zenda

**Affiliations:** Section of Radiation Safety and Quality Assurance, National Cancer Center Hospital East, 6-5-1, Kashiwanoha, Kashiwa, Chiba 277-8577, Japan; Department of Radiological Sciences, Tokyo Metropolitan University, 7-2-10 Higashi-Ogu, Arakawa-ku, Tokyo 116-8551, Japan; Section of Radiation Safety and Quality Assurance, National Cancer Center Hospital East, 6-5-1, Kashiwanoha, Kashiwa, Chiba 277-8577, Japan; Section of Radiation Safety and Quality Assurance, National Cancer Center Hospital East, 6-5-1, Kashiwanoha, Kashiwa, Chiba 277-8577, Japan; Department of Diagnostic Radiology, National Cancer Center Hospital East, 6-5-1, Kashiwanoha, Kashiwa, Chiba 277-8577, Japan; Department of Radiation Oncology, National Cancer Center Hospital East, 6-5-1, Kashiwanoha, Kashiwa, Chiba 277-8577, Japan; Department of Radiation Oncology, National Cancer Center Hospital East, 6-5-1, Kashiwanoha, Kashiwa, Chiba 277-8577, Japan; Department of Radiation Oncology, National Cancer Center Hospital East, 6-5-1, Kashiwanoha, Kashiwa, Chiba 277-8577, Japan; Department of Radiation Oncology, National Cancer Center Hospital East, 6-5-1, Kashiwanoha, Kashiwa, Chiba 277-8577, Japan; Department of Radiological Sciences, Tokyo Metropolitan University, 7-2-10 Higashi-Ogu, Arakawa-ku, Tokyo 116-8551, Japan; Department of Radiation Oncology, National Cancer Center Hospital East, 6-5-1, Kashiwanoha, Kashiwa, Chiba 277-8577, Japan; Department of Radiation Oncology, National Cancer Center Hospital East, 6-5-1, Kashiwanoha, Kashiwa, Chiba 277-8577, Japan

**Keywords:** brachytherapy, treatment planning, dose scaling, tumor control probability, personalized medicine

## Abstract

The purpose of this study was to identify the characteristics of cervical cancer patients who benefit most from dose scaling (DS) using a model-based approach in image-guided adaptive brachytherapy (IGABT). We retrospectively analyzed 57 cervical cancer patients who underwent IGABT and external beam radiotherapy. Model-based DS was simulated to increase the brachytherapy dose until predefined normal tissue complication probability thresholds were reached. Clinically significant therapeutic gain was predefined as a tumor control probability (TCP) increase of greater than 3%, consistent with prior radiobiological modeling studies. Patient characteristics were compared between groups with and without significant TCP gain. Of the 53 patients in whom dose escalation was feasible, 16 (30.2%) achieved a significant TCP gain. A significant gain was strongly associated with histopathological type and magnetic resonance imaging (MRI) findings. All four patients (100%) with adeno/adenosquamous carcinoma (AdSq) achieved a significant gain, compared with only 12 of 49 (24.5%) with squamous cell carcinoma (*P* = 0.006). Uterine corpus invasion (UCI) on MRI was also a significant predictor of benefit (*P* < 0.001). Conversely, larger high-risk clinical target volume limited the potential for dose increase. AdSq cases demonstrated substantial improvement, with TCP increases after DS often exceeding 10%. Patients with poor prognostic features on MRI, specifically UCI, and those with histological AdSq may benefit most from DS in IGABT for cervical cancer. This exploratory study suggests that model-based DS shows promise for personalized treatment planning, supporting a shift from fixed-dose prescriptions toward strategies tailored to individual patient and tumor characteristics.

## INTRODUCTION

The quality of radiotherapy treatment plans is assessed using dose-volume histograms [1]. In brachytherapy combined with external beam radiotherapy (EBRT) for cervical cancer, the cumulative dose is evaluated using equivalent doses in 2 Gy fractions and compared with the planning objectives of predefined dose-volume constraints [2]. When the objectives are not met, decision-making regarding trade-offs between the target dose and dose to the organs at risk (OARs) currently relies on the clinical judgment of radiation oncologists [3]. Such judgments include subjective assessments, leading to potential variability in treatment plan quality among radiation oncologists. Thus, the decision-making process remains a challenge in modern radiotherapy, which is increasingly moving toward personalized medicine [4].

To overcome this limitation, Hoffmann et al. applied the concepts of therapeutic operating characteristic graphs for treatment planning [5]. This approach enables quantitative evaluation of the radiobiological effects by visualizing the relationship between dose–response models such as tumor control probability (TCP) and normal tissue complication probability (NTCP). Ecker et al. applied this model-based approach to brachytherapy combined with EBRT for cervical cancer and validated it with data from 45 patients [6]. After initial treatment planning, they performed uniform dose scaling (DS)—a method where the entire brachytherapy dose distribution is proportionally escalated or de-escalated [6]. They demonstrated that dose escalation using DS improved the TCP by an average of 2% within predefined NTCP limits for nine patients [6].

While Ecker et al. highlighted the potential TCP improvement in illustrative patient scenarios [6], the characteristics of patients who benefit from this model-based approach remain unclear. Although adverse prognostic factors leading to low TCP in cervical cancer have been clarified [7], low baseline TCP does not automatically translate to a large therapeutic gain. This is because the mathematical potential for TCP improvement is clinically realizable only if OAR constraints permit sufficient dose escalation. Therefore, it is crucial to identify specific patient phenotypes that possess the optimal balance of biological need and dosimetric feasibility to transition from fixed-dose prescriptions to stratified, characteristic-based dose prescriptions.

While dose–response models mathematically predict larger TCP gains for patients with low baseline TCP, such gains are clinically meaningful only if sufficient NTCP margin exists to permit dose escalation. Therefore, the primary objective of this study was not to reconfirm mathematical dose–response behavior, but to determine which patient phenotypes can achieve clinically realizable TCP gains under NTCP-constrained dose escalation using a model-based framework. In this study, we retrospectively analyzed cervical cancer patients who underwent image-guided adaptive brachytherapy (IGABT) combined with EBRT and evaluated achievable TCP gains within OAR dose constraints. By integrating radiobiological need (baseline TCP position on the dose–response curve) and dosimetric feasibility (available NTCP headroom), we aimed to establish a clinically actionable patient-selection framework for model-based dose escalation. Because our institutional workflow uses computed tomography (CT)-based contouring whereas models derived from the EMBRACE study, an international study on image-guided brachytherapy in cervical cancer, are based on magnetic resonance imaging (MRI) [8], this study also provides a pragmatic evaluation of the cross-modality applicability of these models in real-world clinical practice. We hypothesized that patients with specific poor prognostic features—such as adenocarcinoma histology or uterine corpus invasion (UCI)—would exhibit an optimal balance between radiobiological demand and dosimetric feasibility, allowing meaningful TCP gains while maintaining acceptable risks of toxicity. The results of this study are intended to inform patient selection strategies for future prospective trials with stratified dose prescriptions. This study contributes to bridging the gap between theoretical dose–response prediction and clinically achievable therapeutic gain under real NTCP constraints.

## MATERIALS AND METHODS

### Patients and treatment

Fifty-seven consecutive cervical cancer patients who underwent IGABT combined with EBRT between June 2021 and January 2024 were included in this study. Treatment planning for EBRT and brachytherapy was performed using Eclipse version 16.1.02 (Varian Medical Systems, Palo Alto, CA, USA) and Oncentra Brachy version 4.6.2 (Elekta AB, Stockholm, Sweden), respectively. For brachytherapy section, radiation oncologists used CT images to delineate the high-risk clinical target volume (CTV_HR_), bladder, rectum, sigmoid colon and small bowel. Medical physicists performed catheter reconstructions and optimized the dose distribution using hybrid inverse planning optimization. Finally, radiation oncologists verified the treatment plan with manual minor modifications of source dwell weights. All patients received EBRT using volumetric modulated arc therapy with a total dose of 45 Gy in 25 fractions, followed by iridium-192 high-dose-rate brachytherapy delivering 24 Gy in four fractions. The cumulative dose of equivalent doses in 2 Gy fractions was calculated using a linear-quadratic model, applying α/β values of 10 Gy for CTV_HR_ and 3 Gy for OARs.

### Dose–response models

In this study, we used the TCP and NTCP models based on Cox proportional hazards regression analysis of the EMBRACE-I trial data [6, 8–10]. The TCP model for 5-year local control incorporated the CTV_HR_ minimum dose covering 90% or less of the volume (D_90%_), tumor histopathological type, CTV_HR_, presence of necrosis and location of UCI at diagnosis on MRI, and overall treatment time (OTT) as covariates. The median CTV_HR_ during four brachytherapy planning sessions was used. The MRI findings were categorized through consensus between a diagnostic radiologist and radiation oncologist. NTCP models used the minimum dose covering 2 cc or less of the volume as covariates to predict Common Terminology Criteria for Adverse Events version 3.0 grade ≥ 2 toxicities in the following three organs: bladder (bleeding, cystitis and fistula), rectum (bleeding, proctitis and fistula) and small bowel (fistula, strictures, incontinence and bleeding). Although the sigmoid colon was delineated as an OAR during the treatment planning, it was excluded from the analysis because a clear correlation has not yet been established between the dose and adverse events [11, 12]. All regression coefficients and model parameters are detailed in Supplement 1. The TCP values for CTV_HR_ and NTCP values for OARs were calculated. For dose summation, EBRT and brachytherapy doses were converted to equivalent doses in 2 Gy fractions (EQD2) using the linear–quadratic model (α/β = 10 Gy for tumor and 3 Gy for OARs) and were summed arithmetically. OTT was not incorporated into EQD2 dose summation; instead, OTT was included as an independent covariate in the TCP model to account for treatment time–dependent tumor repopulation effects.

This study was designed as an exploratory analysis to evaluate the applicability of these models in our CT-based institutional cohort.

### Dose escalation

Dose escalation using DS was performed by proportionally increasing the dose distribution for brachytherapy to increase the TCP until any of the NTCP values reached our institutional limits, as detailed in Table 1. These thresholds were derived from our institutional OAR dose constraints and converted to corresponding NTCP limits using the dose–response models described above. Specifically, the NTCP limits corresponded to physical D_2cc_ constraints of 80 Gy for the bladder, 70 Gy for the rectum, and 70 Gy for the small bowel, based on the ASTRO Clinical Practice Guidelines [13] and established safety thresholds [14]. The scaling factor was defined as the ratio of the post- to pre-scaling prescribed dose. Dose escalation was not performed for patients whose OARs initially exceeded these NTCP limits (scaling factor set to 1.0). Patients were grouped based on whether their dose was escalated (scaling factor > 1.0) or not changed (scaling factor = 1.0). The ΔD_90%_ and ΔTCP were defined as the difference between the post- and pre-DS values for CTV_HR_ D_90%_ and TCP, respectively. A ΔTCP of more than 3% was considered a significant therapeutic gain based on previous studies [15, 16]. Clinically, even a 3–5% improvement in TCP has been regarded as meaningful, as it may translate into tangible increases in long-term local control rates for high-risk subgroups. Thus, even seemingly small percentage changes translate into tangible patient benefits when applied to a cohort with an inherently poor prognosis. Uniform dose escalation was adopted as a pragmatic simplification to enable the systematic evaluation of TCP/NTCP trade-offs. Because DS was applied to clinically generated brachytherapy dose distributions, the actual applicator/catheter geometry and dwell-time distribution were retained. However, DS did not include patient-specific re-optimization of dwell times or modification of applicator/catheter geometry after dose escalation.

**Table 1:** NTCP limits of each OAR for DS

OAR	Index	Constraint (Gy)	NTCP (%)
Bladder	D_2cc_	80	14.36
Rectum	D_2cc_	70	14.32
Small bowel	D_2cc_	70	14.67

D_2cc_, minimum dose covering 2 cc or less of the volume.

### Statistical analysis

Fisher’s exact test was used to compare categorical variables between groups. Post-hoc pairwise comparisons using Bonferroni correction were performed specifically for categorical variables with three or more categories only when the initial omnibus Fisher’s exact test indicated statistical significance. For continuous variables, including analyses of ΔD_90%_ and ΔTCP, data were summarized using medians and interquartile ranges, and group-wise comparisons were performed using the Mann–Whitney U-test. Significant differences between groups were reported with *P*-values where applicable. In visual summaries, medians were represented by horizontal bars, and statistically significant differences were annotated with *P*-values to enhance interpretability. All statistical tests were two-sided and performed using EZR software version 4.3.1 (Saitama Medical Center, Jichi Medical University, Saitama, Japan) [17], with significance set as *P* < 0.05.

To quantify uncertainty in the AdSq subgroup finding, exact 95% confidence intervals (CIs) were calculated for the proportion of patients achieving clinically significant gain (ΔTCP >3%) by histology. Bootstrap resampling was also performed to estimate 95% CIs for the mean difference in ΔTCP between AdSq and SQ. Bootstrap resampling was repeated 5000 times with stratification by histology. As a pragmatic consistency check of the TCP calculation, TCP was recalculated from the model coefficients and baseline hazard and compared with the stored TCP values used in the analysis. For the AdSq–SQ comparison, resampling was stratified by histology. To assess model-parameter sensitivity, each non-zero TCP model coefficient was independently perturbed by ±10% and ±20%, while the other coefficients were held fixed. Mean ΔTCP was then recalculated for the overall cohort and each histological subgroup, and the AdSq–SQ difference was recalculated.

## RESULTS

The baseline characteristics of the 57 patients are summarized in Table 2. These characteristics were categorized according to the factors used in the TCP calculation. The OTT was dichotomized at 50 days, as this was a common threshold in the EMBRACE trials [8, 18, 19]. The study population consisted predominantly of patients with squamous cell carcinoma (SQ; 93.0%), with a smaller group having adeno/adenosquamous carcinoma (AdSq; 7.0%). Most patients (80.7%) had a CTV_HR_ of <30 cm^3^. Necrosis was present in 70.2% of patients, and UCI was observed in 54.4%. The OTT was ≤50 days for 36 patients (63.2%) and >50 days for 21 patients (36.8%). Initial TCP values varied according to the prognostic factors used in the model. In contrast, baseline NTCP values showed limited variation across most subgroups, with the notable exception of the CTV_HR_ category, where patients with larger volumes (> 30 cm^3^) exhibited higher initial NTCPs for the bladder and rectum.

**Table 2:** Baseline patient characteristics and initial TCP/NTCP values

Variables	Number of patients	TCP (%)	NTCP (%)		
			Bladder	Rectum	Small bowel
Histopathological type					
SQ	53	91.0 (86.8–94.5)	8.78 (7.80–10.5)	5.53 (4.47–7.03)	10.7 (7.87–12.1)
AdSq	4	66.2 (63.4–69.5)	7.95 (7.55–8.59)	7.95 (5.31–10.4)	9.72 (8.64–10.2)
CTV_HR_ volume					
<30 cm^3^	46	91.5 (86.9–94.5)	8.53 (7.68–9.72)	5.12 (4.32–6.80)	10.7 (7.93–11.9)
30–45 cm^3^	8	86.7 (83.8–88.4)	10.2 (9.48–12.1)	7.30 (6.74–9.42)	10.6 (7.97–12.4)
>45 cm^3^	3	67.9 (64.3–72.7)	10.8 (9.95–11.0)	11.9 (9.01–13.2)	8.83 (8.23–9.15)
Necrosis at diagnosis on MRI					
Absent	17	95.3 (94.3–96.4)	8.53 (7.68–9.48)	5.53 (4.47–6.88)	9.03 (7.67–11.7)
Present	40	87.4 (83.9–91.0)	8.84 (7.77–11.0)	5.55 (4.53–7.71)	10.7 (8.13–12.1)
UCI at diagnosis on MRI					
Absent	26	94.5 (92.9–96.3)	8.67 (7.71–9.94)	5.33 (4.40–6.73)	10.9 (8.32–11.9)
Lower third	18	87.1 (84.5–88.9)	8.47 (7.74–11.1)	5.63 (4.55–9.97)	9.75 (7.72–11.0)
Middle third	10	84.7 (78.7–86.8)	8.08 (7.69–10.2)	5.40 (4.32–7.48)	9.35 (7.92–10.6)
Upper third	3	77.0 (74.1–81.6)	10.4 (9.96–11.6)	9.11 (7.87–9.81)	12.3 (11.3–13.6)
OTT					
≤50 days	36	90.0 (86.7–94.9)	8.57 (7.65–10.4)	5.45 (4.36–7.00)	10.7 (8.14–12.0)
>50 days	21	91.0 (84.0–92.2)	9.06 (8.19–10.2)	5.53 (4.81–7.83)	9.48 (7.62–11.1)

The dose escalation while respecting the predefined NTCP limits was feasible in 53 of 57 patients (93.0%). For the remaining four patients, the initial plans already exceeded NTCP constraints, precluding any dose escalation. Among the four patients who were ineligible for dose escalation, two exceeded the bladder NTCP (14.76 and 14.94%, respectively), and two exceeded the rectum NTCP (14.63 and 14.83%, respectively).

**Fig. 1:**
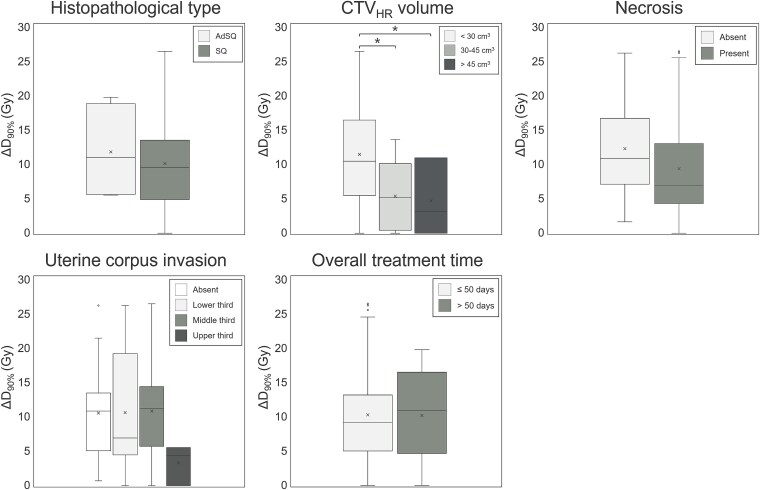
Distribution of ΔD_90%_ stratified by patient characteristics.

Figure 1 illustrates the distribution of the ΔD_90%_, stratified by patient characteristics. While the potential for dose increase was similar across several subgroups, it was significantly limited in patients with a larger CTV_HR_ (> 30 cm^3^) compared with those with a CTV_HR_ of <30 cm^3^ (*P* = 0.04 for 30–45 cm^3^, *P* = 0.03 for >45 cm^3^). Additionally, patients with UCI in the upper third tended to have a smaller ΔD_90%_, although this difference was not statistically significant.

**Fig. 2:**
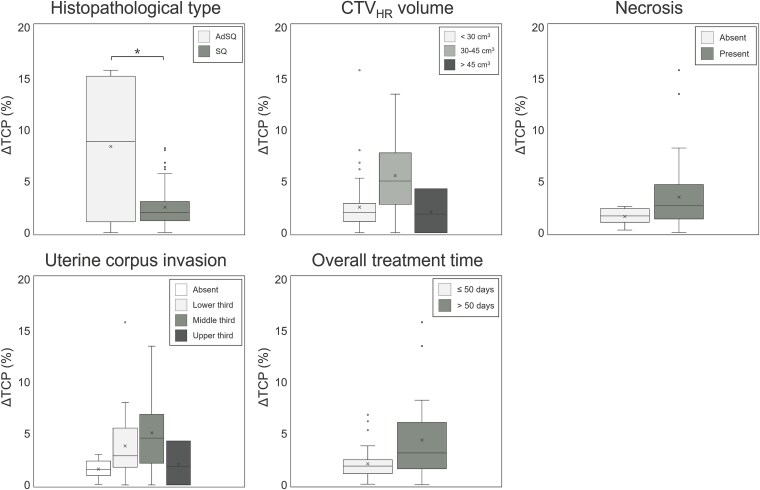
Distribution of the ΔTCP stratified by patient characteristics.

Figure 2 shows the distribution of the ΔTCP after dose escalation. After dose escalation, patients with AdSq and those with UCI (excluding the upper third) demonstrated a significantly greater ΔTCP compared with patients with SQ and those without UCI, respectively.

Table 3 shows comparisons of patient characteristics based on the therapeutic TCP gain. All four patients with AdSq histology achieved a ΔTCP of >3%, compared with only 12 of 37 (32%) patients with SQ (*P* = 0.006). The exact 95% CIs were 39.8–100% for AdSq and 18.0–49.8% for SQ. For ΔTCP as a continuous endpoint, the bootstrap mean difference between AdSq and SQ was 6.9% (95% CI, 1.9–11.9%). In the TCP calculation consistency check, the mean absolute differences between recalculated and stored values were 0.34% for baseline TCP, 0.29% for post-DS TCP and 0.05% for ΔTCP. Furthermore, a significant TCP gain was observed in none of the 26 patients without UCI, but in 8 of 16 (50%) with lower-third invasion and 7 of 9 (78%) with middle-third invasion (*P* < 0.001). These factors were predictive of significant benefit from dose escalation, whereas tumor volume, presence of necrosis and OTT were not. Bootstrap analysis showed that the mean ΔTCP was 2.9% in the overall cohort (95% CI, 2.2–3.7), 2.4% in SQ (95% CI, 1.9–3.0) and 9.3% in AdSq (95% CI, 4.5–14.1). In the parameter perturbation sensitivity analysis, the AdSq–SQ difference in mean ΔTCP remained positive when each non-zero TCP model coefficient was varied by ±10% and ±20%, ranging from 5.5 to 8.5%.

**Table 3:** Comparison of patient characteristics between groups with and without clinically significant TCP improvement (>3%)

Variables	Number of patients	
	ΔTCP >3%	ΔTCP ≤3%
Histopathological type		
SQ	12	37
AdSq	4	0
CTV_HR_ volume		
<30 cm^3^	11	33
30–45 cm^3^	2	4
>45 cm^3^	3	0
Necrosis at diagnosis on MRI		
Absent	2	15
Present	14	22
UCI at diagnosis on MRI		
Absent	0	26
Lower third	8	8
Middle third	7	2
Upper third	1	1
OTT		
≤50 days	10	23
>50 days	6	14

## DISCUSSION

This study is the first to use a model-based approach to identify specific patient subgroups that benefit from dose escalation among patients with cervical cancer treated with combined EBRT and IGABT, challenging the conventional one-size-fits-all dose prescription strategy. Although the overall sample size was modest, the observed effect in the AdSq and UCI subgroups was striking. These results should be regarded as hypothesis-generating and warrant verification in larger multicenter cohorts. Furthermore, the consistency with the findings of the retroEMBRACE trial [20] and EMBRACE-I trial [8] supports the biological plausibility of our results. While Ecker et al. demonstrated the potential of a model-based approach to improve TCP [6], our research pinpoints the precise patient characteristics associated with the most substantial therapeutic gains. This finding is crucial for advancing the field from uniform dose prescriptions toward stratified protocols.

We observed that in patients with large tumor volumes (≥30 cm^3^), the potential for dose escalation was frequently constrained by the NTCP limits of adjacent OARs, as large tumors inherently increase the dose to the bladder and rectum [21]. Furthermore, UCI into the upper third expands the CTV_HR_, resulting in the irradiation of a larger OAR region. This results in higher baseline OAR doses, limiting the achievable TCP gain under an NTCP-limited dose escalation framework. In our cohort, four patients were ineligible for dose escalation for this reason—two because of bladder NTCP limits and two because of rectum NTCP limits. Critically, these are often the patients with lower local control rates who require dose escalation the most [18]. This challenge may be overcome through advanced technical approaches, such as combined intracavitary/interstitial brachytherapy or the use of additional interstitial needles, to deliver a sufficient target dose while respecting OAR dose constraints [18, 22, 23].

Dose escalation was feasible in 53 of 57 patients (93.0%), confirming previous findings that an opportunity for significant TCP improvement exists in most cases [6]. Our analysis revealed that patients with AdSq histology or UCI into the lower two-thirds achieved greater TCP gains with a similar dose increase (ΔD_90%_). These features are established poor prognostic factors that are associated with a lower initial TCP [7], positioning them on the steepest segment of the dose–response curve. AdSq histology is associated with radioresistant tumor biology and lower baseline control rates, while UCI reflects more extensive local spread requiring higher effective doses. These characteristics biologically explain why modest dose increments yield disproportionately large TCP improvements. Consequently, even a modest dose increase yields a disproportionately large improvement in TCP. Therefore, a strategy of aggressive but safe dose escalation is a rational approach to maximize the therapeutic ratio in patients with AdSq histology or UCI below the upper third.

Our study has several limitations. The relatively small sample size, particularly within the AdSq subgroup, limits statistical power and external validity; therefore, these findings should be interpreted as exploratory and hypothesis-generating. However, the observed benefit was consistent across the AdSq subgroup and most UCI cases. Given that these subgroups included only a limited number of patients, particularly the AdSq cohort (*n* = 4), and that the exact CIs and bootstrap resampling indicated wide uncertainty around the AdSq estimate, our results should be regarded as hypothesis-generating evidence. Although the bootstrap CIs and parameter perturbation analysis supported the direction of the AdSq trend, the CIs were wide because of the small AdSq subgroup. These findings should be interpreted with caution but provide a strong rationale for validation in larger multicenter cohorts. These findings are highly consistent with established EMBRACE-I and retroEMBRACE data identifying AdSq histology and UCI as independent adverse prognostic factors. Thus, our results should be regarded as hypothesis-generating evidence that provides a strong rationale for future multicenter validation, rather than as a statistical artifact of small numbers.

The dose–response models were derived from the MRI-based EMBRACE-I trial, whereas our institutional workflow relies on CT for contouring. Although our institution performs CT-based contouring, whereas the EMBRACE-I models were derived from MRI-based volumes, consensus guidelines and comparative studies have demonstrated reasonable consistency between CT and MRI when standardized protocols are applied [24, 25]. Previous studies comparing CT and MRI contouring [26, 27], have reported that while CT tends to overestimate the CTV_HR_ width due to limited soft-tissue contrast, the dosimetric parameters for OARs are generally comparable. Although CT tends to overestimate the tumor width compared to MRI, creating potential uncertainties in absolute TCP values, the relative classification of patient subgroups remains robust against these modality differences. Therefore, the key clinical implication of our study remains robust despite this methodological limitation.

Furthermore, since we applied the EMBRACE-derived model parameters without external validation, some uncertainty may exist regarding the exact slope applicable to our population. However, variation in the model’s slope might affect the absolute values of ΔTCP, it is unlikely to alter the qualitative conclusion that patients with poor prognostic factors (AdSq/UCI) derive disproportionately greater benefit from dose escalation compared to those with favorable features. Importantly, the primary interpretation of our results is based on the relative position of patients along the dose–response curve rather than absolute ΔTCP values, which are more sensitive to model slope assumptions.

Finally, as the models used in our cohort have not been externally validated, this work should be viewed as an exploratory simulation. Future multicenter studies or analyses of institutional outcomes are required to validate these findings. In addition, future research should aim to translate these findings into clinical protocols; this may be done through the design of prospective trials or the development of guideline-based stratified dose-prescription criteria.

Within the limitations of this exploratory study, our findings suggest that prescribed dose escalation may offer a promising avenue to improve outcomes, particularly for patients with AdSq histology or MRI-defined invasion of the lower two-thirds of the uterine corpus. However, given the small sample size, especially in the AdSq subgroup, this strategy requires validation in larger cohorts before clinical implementation.

## Supplementary Material

Supplement_1_rrag047
