# Supplementary material for: Patient stratification for dose scaling in cervical cancer: a model-based analysis using image-guided adaptive brachytherapy
Source: J Radiat Res. 2026 Jul 7;67(4):665–72. doi: 10.1093/jrr/rrag047 (PMC13400573; doi:10.1093/jrr/rrag047)
Supplement: Supplement_1_rrag047 [file supplement_1_rrag047.pdf]

Supplement 1. Cox regression coefficients and model parameters for dose-response models. The parameters are adapted from the supplementary materials of Ecker et al [6].

Endpoint	Covariate	Category	Baseline hazard	Coefficient
5-year local control	Histopathological type	SQ	0.1347	0
		AdSq		1.1750
	CTV_{HR} volume	< 30 cm^3		0
		30-45 cm^3		-0.0453
		> 45 cm^3		0.7778
	Necrosis at diagnosis on MRI	Absent		0
		Present		0.5178
	UCI at diagnosis on MRI	Absent		0
		Lower third		0.6518
		Middle third		0.7416
		Upper third		1.093
	OTT (days)	continuous		0.0329
	CTV_{HR} $D_{90\%}$ (Gy)	continuous		-0.0339
Bleeding, Cystitis, Fistula (Grade ≥ 2)	Bladder D_{2cc} (Gy)	continuous	0.0108	0.0333
Proctitis, Anal/Rectum bleeding (Grade ≥ 2)	Rectum D_{2cc} (Gy)	continuous	0.0008	0.0752
Flatulence (Grade ≥ 2)	Small bowel D_{2cc} (Gy)	continuous	0.0152	0.0335
